# Are all underimmunized measles clusters equally critical?

**DOI:** 10.1098/rsos.230873

**Published:** 2023-08-16

**Authors:** Sifat Afroj Moon, Achla Marathe, Anil Vullikanti

**Affiliations:** ^1^ Network Systems Science and Advanced Computing, Biocomplexity Institute, University of Virginia, Charlottesville, VA, USA; ^2^ Department of Public Health Sciences, University of Virginia, Charlottesville, VA, USA; ^3^ Department of Computer Science, University of Virginia, Charlottesville, VA, USA

**Keywords:** agent-based model, simulation, immunization, anomaly detection, pattern recognition

## Abstract

This research develops a novel system science approach to examine the potential risk of outbreaks caused by geographical clustering of underimmunized individuals for an infectious disease like measles. We use an activity-based population network model and school immunization records to identify underimmunized clusters of zip codes in the Commonwealth of Virginia. Although Virginia has high vaccine coverage for measles at the state level, finer-scale investigation at the zip code level finds three statistically significant underimmunized clusters. This research examines why some underimmunized geographical clusters are more critical in causing outbreaks and how their criticality changes with a possible drop in overall vaccination coverage. Results show that different clusters can cause vastly different outbreaks in a region, depending on their size, location, immunization rate and network characteristics. Among the three underimmunized clusters, we find one to be critical and the other two to be benign in terms of an outbreak risk. However, when the vaccine coverage among children drops by just 5% (or 0.8% overall in the population), one of the benign clusters becomes highly critical. This work also examines the demographic and network properties of these clusters to identify factors that are responsible for affecting the criticality of the clusters. Although this work focuses on measles, the methodology is generic and can be applied to study other infectious diseases.

## 1. Introduction

Measles is a highly contagious vaccine-preventable disease [1]. The United States (US) maintains a very high vaccination coverage level to induce herd immunity. However, disruptions in routine immunizations caused by the COVID-19 pandemic have become a major concern for the heightened risk of outbreaks of vaccine-preventable diseases, in general, and measles, in particular [2–4]. In 2021, 25 million children were estimated to have missed their routine immunizations [5].

According to a recent World Health Organization (WHO) report, measles cases were up by 79% worldwide in the first two months of 2022 compared to the same period in 2021. In 2022, many countries experienced measles outbreaks, such as India, Somalia, Yemen, Zimbabwe and Pakistan. Zimbabwe’s Ministry of Health and Child Care reported more than 6500 cases of measles on 6 September 2022 [6].

In addition, spatial clustering of unvaccinated or underimmunized individuals may increase the risk of outbreaks. For instance, the 2019 measles outbreak in the US shows that spatial concentration of unvaccinated people can cause an epidemic outbreak even when the overall vaccine coverage rate in the region is high enough for herd immunity [7,8]. Several prior studies have demonstrated the existence of spatial underimmunized clusters of the Measles, Mumps and Rubella (MMR) vaccine in the US using different methods [9–11]. A scan statistics method is used in [12,13] to identify statistically significant geographical underimmunized clusters. This hypothesis testing approach for anomaly detection has previously been used in several studies to detect hotspots and anomalies in spatial datasets [14,15].

Most of the prior is only focused on finding underimmunized clusters. However, not all underimmunized clusters pose an equal risk of outbreaks. For instance, although Virginia has a high immunization coverage rate of 95.8% among kindergartners [16], which is more than the target immunization rate for herd immunity, it has multiple underimmunized clusters, as shown in this paper. Further, Virginia has a high importation risk because of the two major international airports. For instance, a measles outbreak in 2021 affected the Central and Northern Health Regions of Virginia [17]. Additionally, as mentioned above, COVID-19 has disrupted routine immunizations, which might have led to a drop in MMR immunization rates across the state. The only way to estimate the actual risk due to an underimmunized cluster is to consider disease transmission.

**Figure 1.**
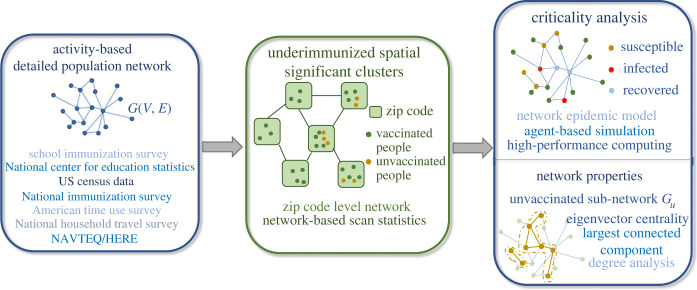
Four major components of the framework: (i) an activity-based population network *G*(*V*, *E*), where a node represents an individual, and an edge represents a contact between two people; (ii) underimmunized spatial clusters in a zip code level network *G*_*z*_(*V*_*z*_, *E*_*z*_), where nodes are zip codes, and a connection between two zip codes represents a geographically shared boundary; (iii) criticality analysis of each cluster using the stochastic network epidemic model and (iv) understanding criticality by investigating network properties of unvaccinated sub-network *G*_*u*_.

In this research, we formalize the outbreak risk of a cluster by its ‘criticality’, which is defined as the ‘probability of a large outbreak’ caused by a single case of measles in the cluster. We focus on clusters that are most significant in terms of underimmunization rates and measure their criticality. Finding significant underimmunized clusters and computing their criticality is a challenging computational problem. We use a synthetic social contact network model for Virginia, and school-level immunization data in the state, along with a network scan statistics approach to find significant underimmunized clusters. We combine this with a detailed stochastic agent-based simulation framework to estimate the criticality of each significant cluster, by simulating outbreaks that originate in these clusters (figure 1).

We also examine how the criticality of clusters changes under a hypothetical 5% drop in MMR rate among children (younger than 12 years), possibly due to disruptions caused by COVID-19. Finally, we study the demographic, geographical and network factors associated with such clusters, which can help explain the potential risk of a cluster. Identifying critical clusters and the factors associated with them will be of use to public health authorities who can use this information to prioritize mitigation efforts.

Note that although this work focuses on measles, as it uses MMR coverage data and a measles-specific disease model, the methodology is generic and can be applied to study other infectious diseases.

## 2. Methods

To find significant clusters and their criticality, we first identify statistically significant underimmunized clusters; for this purpose, we develop a zip code level spatial network from a detailed activity-based population contact network and use a network scan statistics method. Next, we investigate the criticality of each cluster by importing a single case of measles and simulating its spread using an agent-based model.

### 2.1. Synthetic social contact network model

We construct a contact graph *G*(*V*, *E*) for a population *V*, on which a disease can spread. Nodes *v* ∈ *V* represent individuals (referred to as ‘nodes’), and an edge (*u*, *v*) ∈ *E* represents a contact between two nodes *u* and *v*, on which disease can spread. Each edge in the network *G* is associated with a weight that is equal to the duration of the contact. Additionally, we assume that each node *v* is associated with a geographical location, denoted by loc(*v*). The nodes in *G* have specific node properties, such as geographical location, age, gender, income, etc. This model combines various datasets from commercial and public sources, including the US census data, into a common architecture for creating a digital twin of the Virginia population. For more details on the construction of the social network, please see our earlier works [9,18–20].

The model places each individual in a household with others, and each household is located geographically in such a way that the aggregation of this synthetic population at the block group level is statistically equivalent to the US census block group data. The daily activities of individuals are assigned using time use surveys such as American Time Use Survey data [21] and National Household Travel Survey Data [22]. The geographical location for each activity is assigned from detailed land use data [23–26]. This activity-based network model also contains information about school locations, which are used for assigning schools to school-aged children [9].

### 2.2. Finding significant underimmunized clusters

The goal here is to identify significant underimmunized spatial clusters. Detailed zip code level immunization rates are not available, but are available at the level of schools. We describe our method for determining zip code level immunization rates, and then finding significant contiguous clusters.

In order to capture spatial contiguity, we consider a zip code level spatial network, *G*_*z*_(*V*_*z*_, *E*_*z*_) for Virginia, where nodes are zip codes. We note that the term zip code refers to a ZIP Code Tabulation Area (ZCTA), which is an aerial representation of a United States Postal Service (USPS) ZIP Code service area published by the US Census Bureau [27]. The edges in *G*_*z*_ are connections between two zip codes or ZCTAs. If two zip codes share a geographical boundary, they form an edge in the network *G*_*z*_. We use the *G*_*z*_ network to find clusters consisting of geographically contiguous underimmunized zip codes. Each node or zip code *i* has two properties: (i) population size *P*_*i*_, and (ii) immunization rate *r*_*i*_. We get *P*_*i*_ and *r*_*i*_ by aggregating the activity-based synthetic population network model *G* at the zip code level. Finally, we use a modified Kulldroff’s scan statistics method to find statistically significant underimmunized clusters in the network *G*_*z*_ [9].

#### 2.2.1. Modelling immunization rate among children

The goal here is to determine an immunization probability for each node *v* ∈ *V* corresponding to a child. We use the publicly available Virginia school immunization survey (SIS) record [28] for Fall 2018 to calculate the MMR immunization rate among children (up to 17 years old). SIS contains immunization records for public schools. However, few kindergarten-level schools or schools with less than ten students report an overall vaccination rate instead of MMR-specific immunization rate; and for some schools, there is no MMR immunization rate available in the SIS data. To handle the missing MMR immunization rate for these schools, we make the following assumptions:

(i) If the MMR vaccination rate is not available for a school, but the overall immunization rate is available, then we use the overall immunization rate for that school.
(ii) In the synthetic population network model *G*, if the immunization rate for a school is not available from SIS data, we use the nearest neighbouring school’s MMR immunization rate.

We find the nearest school by calculating the Haversine distance using the latitude and longitude. For children younger than 11 years old, we use the corresponding school’s kindergarten immunization record. To infer the immunization rate among chidlren of 12–17 years old, we use the associated school’s 6th-grade immunization record. Previous studies have found that immunization status among children in the same household, even in the neighbourhoods, schools, or jurisdictions, are positively correlated because of the geographical aggregation of vaccine refusal [10,29,30].

#### 2.2.2. Immunization rate among adults

CDC’s ChildVaxView program reports state-level vaccination coverage among 19–35-month-olds via National Immunization Survey [31,32]. To obtain the state-level immunization rate among adults, we use a weighted average of the rates from the National Immunization Survey report for 1995–2004 since the children from these years are adults in the current study period. We assign these immunization rates to the adults uniformly per age group.

#### 2.2.3. Identifying underimmunized clusters using network scan statistics

The previous steps lead to an immunization probability for each individual *v* ∈ *V*; this can be translated to an immunization rate for each zip code *i* ∈ *V*_*z*_. In order to consider clusters without any constraints on their shape, we use the techniques of network scan statistics—these are based on hypothesis testing, and find statistically significant underimmunized clusters or hotspots in network *G*_*z*_ [14,33]. A cluster *C* ⊂ *G*_*z*_ in the proximity network *G*_*z*_ can have an arbitrary shape. We calculate the score function or scan statistic of a cluster of zip codes *C* as *F*(*C*) = *Pr*[Data|*H*_1_(*C*)]/[Data|*H*_0_] which is a likelihood ratio of the probability of the observed data (i.e. a certain level of underimmunization in *C*) generated under an alternative hypothesis *H*_1_(*C*), to the probability of the observations under the null hypothesis *H*_0_. We use the Poisson version of the Kulldorff scan statistic, which assumes that the observations are generated from a simple parametric distribution, Poisson distribution (a common assumption in epidemiological data analysis). The null hypothesis *H*_0_ is generated proportionally from the baseline count (1 − *μ*)*P*_*i*_, where *μ* is the state-wide immunization rate. The alternative hypothesis of a cluster *H*_1_(*C*) counts the vaccine distribution among nodes outside *C*; in *V*_*z*_ − *C*, the unvaccinated count comes from a rate proportional to the baseline counts. But, for the nodes within *C*, the counts are generated at a higher rate than expected. The objective is to find clusters that maximize the scan statistic *F*(*C*). We use the Monte Carlo sampling approach to compute the *p*-value for each cluster. Optimization over arbitrarily shaped clusters is computationally expensive as the score function *F*(*C*) of interest is typically non-convex and NP-hard to optimize. We use a general dynamic programming method for optimizing a large class of parametric and non-parametric scan statistics [34].

### 2.3. Criticality analysis

We describe the notion of criticality formally here, and our methodology for estimating it using a stochastic agent-based network epidemic model, which allows us to investigate the impact of an underimmunized cluster.

#### 2.3.1. Network epidemic model

We use an SEIR model for measles [35–38], where an unvaccinated node can be in one of four health states: susceptible (S), exposed (E), infected (I) and recovered/removed (R). Let **x**(*t*) be the health state vector at time t; *x*_*i*_(*t*) ∈ [0, 1, 2, 3] is the health state of an unvaccinated node *i* at time *t*. Here, 0, 1, 2 and 3 correspond to susceptible, exposed, infected and recovered health state. A node *i* in network G has a vaccination status: vaccinated or unvaccinated. Let **v** be a *vaccination* vector: *v*_*i*_ ∈ [0, 1] denotes the probability that node *i* is vaccinated.

We assume that the MMR vaccine has 100% efficacy [39] (although it may be a little less in practice); therefore, the vaccinated nodes do not directly participate in disease transmission. The presence of vaccinated nodes is important in the model as they affect the pathways to transmission by fragmenting the contact network.

**Figure 2.**
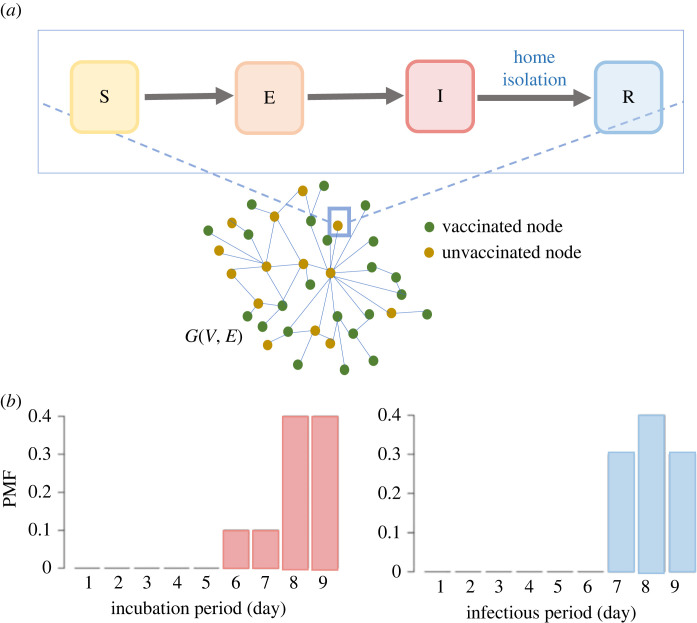
Disease transmission model. (*a*) Network SEIR (susceptible-exposed-infected-recovered) epidemic model, (*b*) probability mass function (PMF) for incubation period and infectious period.

We generally assume the source of the outbreak is a random infection in a subset *V*_*c*_ ⊂ *V*. Here, *V*_*c*_ is the set of nodes which are located in a cluster *C*. We use *I*(**v**, *V*_*c*_) to denote the total number of infections given the vaccination vector **v** and source in *V*_*c*_. We use an agent-based stochastic individual network model to simulate the spread of measles in the contact network *G* (figure 2*a*). This model keeps track of disease progression and different health states of each node.

We assume that initially all the unvaccinated nodes are susceptible, and the disease starts from a random susceptible node. An infected node will transmit the disease to its susceptible neighbours in the contact network *G* with a transmission probability *β*. We use *β* = 0.5, as estimated from the recent New York City (NYC) outbreak that resulted in 649 cases between September 2018 and August 2019 [20]. The outbreak size was calibrated for Virginia’s population size.

The disease model assumes that an exposed node will be infectious after a latent period. The maximum duration of the latent period is nine days and follows a discrete probability distribution {0, 0, 0, 0, 0, 0.1, 0.1, 0.4, 0.4}. The infectious or recovery period of an infected node is also nine days and follows a discrete probability distribution {0, 0, 0, 0, 0, 0, 0.3, 0.4, 0.3} (figure 2*b*) [40]. We assume that a recovered individual will not get reinfected.

#### 2.3.2. Intervention

We assume that 90% (compliance rate) of the new infectious nodes will begin home-isolation after three days as the measles rash starts after 3–5 days [40]. In our network model, all non-home edges of the isolated cases are removed so they can only spread the disease to their household members.

#### 2.3.3. Disease spread

An agent-based stochastic epidemic simulator *EpiHiper* [41,42] simulates the spread of measles in the contact network *G*. It is a scalable system. Parallel programming and distributed memory systems allow it to handle millions of nodes and billions of edges. The simulation starts from a random infected node and runs for 365 time steps (or days). From 300 stochastic *EpiHiper* realizations, we compute the probability of having a large outbreak. If the number of infections exceeds *OC* = 500, we define it as a large outbreak. The total number of infections is $\sum_{t=0}^{365}(x{\left(t\right)}_{1\to 2})$; here, **x**(*t*)_1→2_ is the new infected nodes at day *t*.

#### 2.3.4. Criticality of a cluster

We define the **criticality** of the set *V*_*c*_ ⊂ *V* in immunization **v**, denoted by crit(*V*_*c*_, **v**), as the probability of a large outbreak, when the initial infection occurs in subset *V*_*c*_. Formally, $\mathrm{crit}(V_{c},v)=\mathrm{Pr}[I(v,V_{c})>OC]$, where *OC* is the threshold for a large outbreak (taken to be 500, as mentioned above). We use the probability of an outbreak instead of the more commonly used metric of expected number of infections because the expectation is quite small, as many outbreaks die out due to the high immunization rates.

#### 2.3.5. Characterizing critical clusters

This research hypothesizes that all underimmunized clusters are not equally critical. When seeded, some clusters cause a large outbreak while others do not. To understand the potential reasons behind the criticality of clusters, we investigate their geographical and network properties. We explore the impact of geographical location, size, population density and underimmunization rate on the criticality of a cluster. For network attributes, we measure their degree, node strength and eigenvector centrality. We also investigate the connected components.

#### 2.3.6. Degree

The degree of a node *i* in a network is the number of neighbours of node *i*. If degree of a node *i* is *k*_*i*_, then average node degree of the network is $\sum_{i=0}^{\left|V\right|}k_{i}/\left|V\right|$. The average degree is important as it indicates the connectivity in a network [43]. Moreover, high-degree nodes can act as hubs in spreading the disease [44].

#### 2.3.7. Node strength

The node strength of a node *i* is the strength of the nodes or sum of the weights of the edges connected to it [45].

#### 2.3.8. Eigenvector centrality

It indicates the influence of a node in a network. The eigenvector centrality of a node is proportional to the sum of the centrality of its neighbours. It represents the spectral properties of the adjacency matrix *A* [46]. In contrast to degree centrality, eigenvector centrality takes the entire network into account. This property makes eigenvector centrality particularly useful for understanding the influence of graph characteristics on epidemic spreading [47]. Eigenvector centrality *e*_*i*_ of a node *i* in a network *G*(*V*, *E*) is

$$e_{i}=\lambda_{1}^{-1}\sum_{\;j\in V}a_{ij}e_{j}.$$

Here, *A* = [*a*_*ij*_] is the adjacency matrix of the network *G*, where *a*_*ij*_ = 1 if node *i* has a connection with node *j*, and *a*_*ij*_ = 0 otherwise. *λ*_1_ is the largest eigenvalue or spectral radius of the adjacency matrix *A* [48]. The principal eigenvector, which corresponds to the largest eigenvalue, controls the structural and dynamical properties of a complex network. A large eigenvector centrality of a node indicates that it has many neighbours or important neighbours in the network. Eigenvector centrality is a good measure of a node’s spreading power [49–51].

We use two networks for network analysis, namely, the full contact network *G*(*V*, *E*), and unvaccinated sub-networks *G*_*u*_(*V*_*u*_, *E*_*u*_) ⊂ *G*(*V*, *E*). In a sub-network *G*_*u*_, all the nodes *V*_*u*_ are unvaccinated; analysis of this sub-network is essential as the disease spreads only over this sub-network.

## 3. Results and discussion

### 3.1. Underimmunized clusters in Virginia

According to the CDC immunization record for the 2019–2020 school year, MMR vaccine coverage among kindergartners in Virginia is around the target rate of 95% for herd immunity [52]. In our synthetic Virginia population network, the MMR immunization rate as estimated from various data sources is 96.331% among children and 91.496% for the overall population. We call the estimated immunization vector the base immunization. The synthetic Virginia population contact network *G*(*V*, *E*) has 7 688 058 nodes and 371 888 622 edges; here, nodes are individual people. The zip code network *G*_*z*_(*V*_*z*_, *E*_*z*_) has 892 nodes and 2653 edges; here, a node represents a zip code. For each zip code, we calculate population and immunization rate by aggregating contact network *G*.

**Figure 3.**
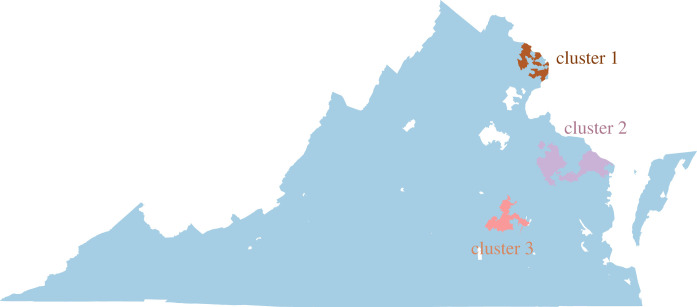
Critical underimmunized clusters in Virginia for MMR (measles, mumps and rubella) vaccine.

Although the MMR coverage is fairly high, the network-based scan statistics method finds three significant underimmunized clusters in Virginia (figure 3). The vaccination rate in these regions varies from 88.2% to 90.6%. The statistical significance of these three clusters is measured from Monte Carlo Simulation: cluster 1 (log-likelihood score: 730.82, *p*-value: 9.99 × 10^−4^) is the most significant one, then cluster 2 (log-likelihood score: 141.01, *p*-value: 9.99 × 10^−4^) and cluster 3 (log-likelihood score: 98.10, *p*-value: 9.99 × 10^−4^).

Table 1 summarizes the geographical and network properties of the three clusters. The first cluster *C*_1_ is located in the highly populated part in northern Virginia. Population density per square mile in cluster 1 is 2684.2, which is over 10 times the average population density per square mile in Virginia (≈202.6). This cluster contains 28 zip codes and is the largest underimmunized cluster in Virginia, with an immunization rate of 90.1%.

**Table 1.** Characteristics of the three statistically significant underimmunized clusters or hotspots in Virginia.

characteristic	cluster 1	cluster 2	cluster 3
geographical location	northern Virginia	middle peninsula of Virginia	Richmond (capital) region of Virginia
no. of zip codes	28	13	9
population count	610 464	24 302	190 832
underimmunized percentage	9.9%	11.8%	9.4%
population density per square mile	2684.2	45.43	799.2
degree in *G*	mean 47.9	mean 33.9	mean 40.8
	median 42	median 28	median 36

Thirteen zip codes in the middle peninsula of Virginia form the second cluster *C*_2_. Although the population density in cluster 2 is only 45.43, which is less than one-fourth of Virginia’s average population density, its immunization rate is the lowest too, at only 88.2%. The second largest cluster, cluster 3, is located in the Richmond region of Virginia. The population density in cluster 3 is about four times higher than in Virginia.

These three clusters are unique concerning geographical location, shape, size, underimmunization rate, population density and network characteristics. Even though the second cluster is the smallest and only affects a small population compared to the other two significant clusters, it can play a crucial role in disease spreading because of its position in the network and a low immunization rate.

### 3.2. Criticality of clusters

To calculate the criticality of a cluster *C*, we simulate an outbreak over the Virginia contact network *G*(*V*, *E*) using our agent-based simulation framework *EpiHiper*. The criticality of a cluster *C*_*i*_ is measured by estimating the probability of getting a large outbreak (similar to the NYC measles outbreak in 2019) if the epidemic starts from a random node in cluster *C*_*i*_. The seed is selected randomly from the unvaccinated age group of 5–17 years old in the cluster.

**Figure 4.**
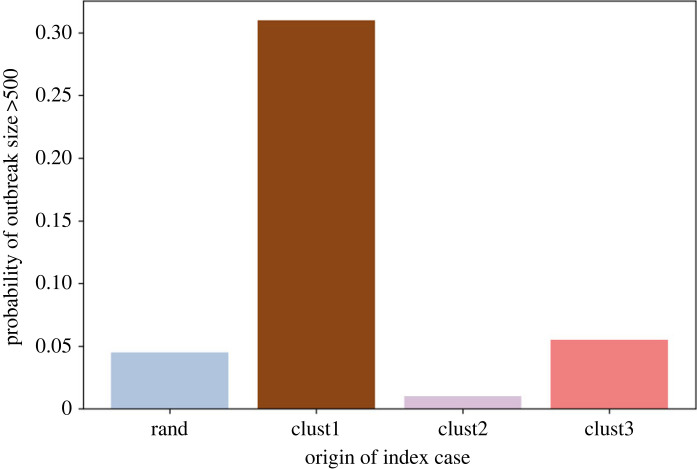
Probability of a large outbreak in Virginia in the base immunization for four different seeding scenarios: (i) random seeding in Virginia (rand), (ii) seeding in cluster *C*_1_ (clust1), (iii) seeding in cluster *C*_2_ (clust2) and (iv) seeding in cluster *C*_3_ (clust3).

We apply four different seeding scenarios: (i) seeding randomly in Virginia (rand), (ii) seeding in cluster *C*_1_, (iii) seeding in cluster *C*_2_ and (iv) seeding in cluster *C*_3_. The epidemic always starts from a single seed. For each scenario, we run 300 replicates and report the aggregated result. The probability of a large outbreak for these four seeding scenarios is presented in figure 4. Our criticality analysis shows that the first cluster is the most critical, where seeding results in a large outbreak in 30% of the instances. By contrast, a random seeding in Virginia (rand) produces a large outbreak in less than 5% instances. Cluster 1 is the largest cluster and has the highest population density and node degree (table 1).

Figure 4 shows that the criticality of cluster 2 is even lower than ‘rand’. The underimmunized pocket in cluster 2 does not increase the probability of large outbreaks, even though cluster 2 has the highest percentage of underimmunized individuals. Cluster 3 has a higher chance of causing an outbreak than ‘rand’ but it is only marginally higher than ‘rand’, unlike cluster 1.

### 3.3. Risk analysis of drop in immunization rate

Due to COVID-19, routine childhood immunizations have been disrupted. To understand its impact on the criticality of the underimmunized clusters, we study a hypothetical scenario where a random uniform 5% drop in MMR immunization rate occurs among age group 12 years or younger in Virginia. The 5% drop in immunization rate among children (12 years or younger) in the State reduces the vaccine coverage in the network *G* by only 0.8%.

**Figure 5.**
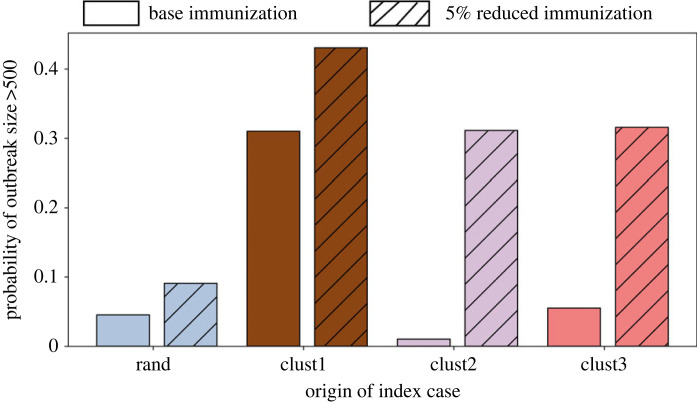
Probability of a large outbreak in Virginia under the base case and the reduced immunization case for the four different seeding scenarios.

Figure 5 presents the probability of a large outbreak for different seeding scenarios under the reduced immunization coverage. We find that the criticality of cluster 1 in the reduced immunization scenario $\mathrm{crit}(V_{C_{1}},r)$ increases by 1.45 times its original criticality in the base case $\mathrm{crit}(V_{C_{1}},b)$. However, the criticality of the second cluster increases by 32.5 times of $\mathrm{crit}(V_{C_{2}},b)$ and the criticality of the third cluster increases by six times of $\mathrm{crit}(V_{C_{3}},b)$. Only a 5% drop in immunization rate among kids (age 12 years or younger) makes the two benign clusters very critical. In the base case, the majority of the epidemic stays inside the cluster. For cluster 1, cluster 2 and cluster 3 seeding, the expected total incidence inside the cluster in base case is 67%, 77% and 76%, respectively (table 2). In the 5% reduced immunization, the expected total incidence inside cluster for cluster 1, cluster 2 and cluster 3 seeding scenarios decrease to 37%, 3.6% and 25.4%, respectively, and increases the incidence outside the cluster. This is not surprising since many new nodes and connections are now part of the *G*_*u*_ network. However, the expected number of cases outside the cluster *C*_2_ go up significantly. For *C*_1_, *C*_2_ and *C*_3_, reduced immunization increases outside-cluster incidence by 40 times, 267 times and 131 times, respectively. To understand possible underlying factors, we examine the network properties of the clusters.

**Table 2.** Size of the outbreak inside and outside clusters under base immunization and reduced immunization rates.

	base immunization		reduced immunization		reduced immunization/base immunization	
seeding	inside cluster	outside cluster	inside cluster	outside cluster	inside cluster	outside cluster
cluster 1	1013.67	305.03	6987.99	12106.32	6.89	39.68
cluster 2	67.28	20.06	198.18	5357.66	2.95	267
cluster 3	60.88	19.11	854.48	2511.49	14.03	131.42

### 3.4. Network analysis of the unvaccinated sub-network *G*_*u*_

In the base immunization case, the unvaccinated sub-network *G*_*u*_ has 653 811 nodes and 2806 876 edges, as shown in table 3. The largest connected component has 525 586 nodes, i.e. more than 80% of the nodes are a part of this giant component in *G*_*u*_.

**Table 3.** Network analysis of the unvaccinated sub-network *G*_*u*_ in the base immunization case and the reduced immunization case. Percentage change quantifies the change in network property due to reduced immunization rate.

features of *G*_*u*_	base immunization	reduced immunization	percentage change
no. of nodes	653 811	714 164	9.20%
no. of edges	2806 876	3251 182	11.87%
largest connected component size	525 586	588 019	11.88%

Table 4 presents six different network properties of each geographical clusters in the unvaccinated sub-network *G*_*u*_, under base rate and reduced immunization rate:

(i) Number of nodes in a cluster *C* in the network *G*_*u*_ or number of unvaccinated nodes locates in cluster *C*.
(ii) Average degree of the nodes of a cluster *C* in *G*_*u*_.
(iii) Average node strength of the nodes of a cluster *C* in *G*_*u*_.
(iv) Number of connections inside of a cluster *C* in *G*_*u*_.
(v) Number of connections from inside of a cluster *C* to outside in *G*_*u*_.
(vi) Average eigenvalue centrality of the nodes of a cluster *C* in *G*_*u*_.

Among the six properties, the first five are local properties, as the full network is not required to calculate them. However, eigenvalue centrality is a global property as it considers the entire network.

**Table 4.** Network properties of the unvaccinated sub-network within each cluster in the base immunization and reduced immunization. Percentage change quantifies the change in network property due to reduced immunization rate.

features	cluster	base immunization	reduced immunization	percentage change
no. of nodes	*C*_1_	60 455	64 786	7.2%
	*C*_2_	2874	2998	4.3%
	*C*_3_	18 019	19 391	7.6%
average degree	*C*_1_	5.31	5.70	7.1%
	*C*_2_	4.83	5.08	5.2%
	*C*_3_	4.10	4.43	8.3%
average node strength	*C*_1_	47 845.88	52 743.10	10.2%
	*C*_2_	53 753.20	57 044.82	6.1%
	*C*_3_	32 140.66	36 373.49	13.2%
no. of connections inside	*C*_1_	168 428	202 236	20.0%
	*C*_2_	8974	10 016	11.6%
	*C*_3_	29 450	37 904	28.7%
no. of connections from inside to outside	*C*_1_	152 840	167 809	9.8%
	*C*_2_	4905	5271	7.5%
	*C*_3_	44 358	48 882	10.2%
average eigenvector centrality	*C*_1_	5.4 × 10^−4^	5.68 × 10^−4^	5%
	*C*_2_	7.73 × 10^−8^	1.62 × 10^−7^	109.7%
	*C*_3_	6.43 × 10^−8^	7.96 × 10^−8^	23.6%

In the base immunization case, cluster *C*_1_ has 60 455 unvaccinated nodes which is 9.2% of the total unvaccinated nodes. The average degree and the average eigenvector centrality in *G*_*u*_ are also very high for this largest cluster. The smallest cluster *C*_2_ has 2874 unvaccinated nodes, which is only 0.4% of the total unvaccinated population.

The 5% drop in immunization rate among children has a significant impact on the network properties of the three clusters. The last column in table 4 shows the percentage change in the value of each feature within each cluster’s unvaccinated network. The unvaccinated network in cluster *C*_3_ shows the maximum change in percentage of all features, except for eigenvalue centrality, compared to the other two clusters’ networks. On the other hand, the unvaccinated network in cluster *C*_2_ shows the minimum change in all features except for eigenvalue centrality.

Although a drop in immunization rate adds only a few new connections from cluster 2 to the outside network, these new connections add critical neighbours to the nodes of *C*_2_ and make it a core part of the unvaccinated network. The average eigenvector centrality of *C*_2_ goes up by 109.7%, which is significantly more than the increase in the other two clusters. This centrality plays a key role in explaining the change in criticality of cluster *C*_2_. As shown earlier in figure 5, the criticality of cluster *C*_2_ increased by 32.5 times in the reduced immunization case.

## 4. Conclusion

This research shows that all underimmunized clusters are not equal in terms of their risk in causing a large outbreak. Some are significantly more critical than others and even a small disruption in routine MMR immunization can disproportionately change the risk of a cluster. This can occur despite a very high immunization rate overall [53]. We develop a system science model to find underimmunized MMR clusters in Virginia and measure their criticality. Although this research is focused on measles, our framework is general enough to estimate criticality of underimmunized clusters for other infectious diseases in other geographical locations.

Our network-based Kulldorff’s spatial scan statistical method finds three significant irregular-shaped underimmunized clusters of zip codes in Virginia. These three clusters have very different geographical locations, sizes, population densities, immunization rates and network properties (table 1). The first cluster *C*_1_ is the largest one and is in a densely populated urban region. It also has a high average node degree and high average eigenvector centrality. The second cluster *C*_2_ is in a rural region. It is the smallest; however, it has the highest underimmunized percentage of individuals. The third cluster *C*_3_ is a large cluster, located near Richmond with a high population density.

To understand the criticality of a cluster, we use a stochastic individual-based network epidemic model that accounts for heterogeneous contacts and detailed immunization information. Our model considers vaccination status and home isolation as interventions at the individual level. The simulation results find that the criticality of underimmunized clusters are different. In the base case, the most critical cluster is the largest cluster *C*_1_. The probability of a large outbreak from seeding in cluster *C*_1_ is more than six times higher than the random seeding in Virginia. On the other hand, the criticality of the second significant cluster *C*_2_ is very low, even lower than the random seeding in Virginia. However, this benign cluster becomes very critical when MMR immunization rate drops by 5% among the age group of 12 years or younger. Currently, this is a major concern since the COVID-19 pandemic has disrupted routine immunization programs globally. According to CDC, more than 61 million doses of measles vaccines were postponed or missed because of the COVID-19 pandemic. In 2022, many countries around the world, including India, Yemen and Somalia suffered measles outbreaks [54].

We find that a 5% immunization drop among children increases the criticality of all clusters, but the percentage increase in criticality is significantly different for different clusters. For example, the criticality of the second cluster increases by 32.5 times the base immunization after the drop in vaccination rate. By contrast, criticality for the most critical one, *C*_1_, increases only 1.45 times, and criticality for the *C*_2_ increases only six times. Reduced immunization rate also changes the incidence rates inside and outside of a cluster. In the 5% reduced immunization scenario, when *C*_2_ is seeded, the number of infections outside the *C*_2_ cluster is about 267 times the number of infections outside the cluster in the base immunization case, while the cases inside the cluster increase to only 2.95 times the base immunization case, which indicates that it was an isolated cluster in the base immunization. However, a small immunization drop among children adds it to the core of the network and increases its outbreak risk significantly.

The network analysis of the unvaccinated network reveals an important direction for public health. We find that the local network properties of a cluster are not enough in measuring its potential risk of an outbreak from a drop in immunization rate. We have considered five local properties: size, degree, node strength, links inside of a cluster and connections inside to outside. A small drop in immunization coverage (only 0.8%) increases the outbreak risk of *C*_2_ the most; however, surprisingly, the increment in local properties of cluster *C*_2_ is the lowest among the clusters (table 4, rows 1–5). We find that while the local properties can be misleading, eigenvector analysis can provide a better understanding of the change in criticality of a previously benign cluster, *C*_2_, as it considers the full network connectivity. This research shows that the outbreak risk of underimmunized clusters is vastly different depending on their location, size, immunization rate and network properties.

## Data Availability

This study uses retrospective data available along with computer code at https://github.com/SAfrojM/Criticality-of-a-cluster.
